# Simulation Design of an Elliptical Loop-Microstrip Array for Brain Lobe Imaging with an 11.74 Tesla MRI System †

**DOI:** 10.3390/s25134021

**Published:** 2025-06-27

**Authors:** Daniel Hernandez, Taewoo Nam, Eunwoo Lee, Yeji Han, Yeunchul Ryu, Jun-Young Chung, Kyoung-Nam Kim

**Affiliations:** 1 Neuroscience Research Institute, Gachon University, Incheon 21988, Republic of Korea; 2 Department of Health Sciences and Technology, GAIHST, Gachon University, Incheon 21999, Republic of Korea; 3 Department of Biomedical Engineering, Gachon University, Seongnam 13120, Republic of Korea; 4 Department of Radiological Science, Gachon University, Incheon 21999, Republic of Korea; 5 Department of Neuroscience, College of Medicine, Gachon University, Incheon 21565, Republic of Korea

**Keywords:** electromagnetic simulations, magnetic fields, microstrip, MRI

## Abstract

Magnetic resonance imaging (MRI) is a powerful medical imaging technique used for acquiring high-resolution anatomical and functional images of the human body. With the development of an 11.74 Tesla (T) human MRI system at our facility, we are designing novel radiofrequency (RF) coils optimized for brain imaging at ultra-high fields. To meet specific absorption rate (SAR) safety limits, this study focuses on localized imaging of individual brain lobes rather than whole-brain array designs. Conventional loop coils, while widely used, offer limited |B_1_|-field uniformity at 500 MHz—the Larmor frequency at 11.74 T, which can reduce image quality. Therefore, it is important to develop antennas and coils for highly uniform fields. As an alternative, we propose an elliptical microstrip design, which combines the compact resonant properties of microstrips with the enhanced field coverage provided by loop geometry. We simulated a three-element elliptical microstrip array and compared its performance with a conventional loop coil. The proposed design demonstrated improved magnetic field uniformity and coverage across targeted brain regions. Preliminary bench-top validation confirmed the feasibility of resonance tuning at 500 MHz, supporting its potential for future high-field MRI applications.

## 1. Introduction

Magnetic resonance imaging (MRI) is an imaging modality used to acquire anatomical and functional images of the body. MRI has been employed in clinical practice to diagnose various diseases, tumors, cancers, and metabolic disorders [1,2,3]. Acquiring images with MRI requires different types of magnetic fields. The main magnetic field, B_0_, is a strong magnetic field typically used clinically at strengths ranging from 1.5 to 7 Teslas (T). B_0_ aligns the proton spins in the direction of the *z*-axis, with the spin frequency determined by the Larmor frequency, which is proportional to the B_0_ field. Stronger B_0_ fields result in a higher signal as more spins align with the B_0_ field direction [4]. Consequently, there is significant interest in developing MRI scanners with strong B_0_ fields, such as the 11.74 T scanner for human imaging [5]. In addition to the B_0_ field, MRI uses radiofrequency (RF) magnetic fields to tip net magnetization away from the *z*-axis in order to generate detectable signals [6]. Therefore, it is essential to design RF coils capable of generating strong and uniform RF magnetic fields—referred to as the |B_1_|-field —at the corresponding Larmor frequency [7].

The Larmor frequency (*f*) is defined by the following equation:

(1) $$f\;=\;\frac{\gamma B_{0}}{2\pi }$$

where γ is the gyromagnetic ratio, a constant specific to each nucleus. For hydrogen, γ is approximately 42.58 MHz/T, yielding a Larmor frequency of about 500 MHz at 11.74 T. The RF magnetic field used in MRI, denoted as B_1_, is an oscillating field composed of the x and y components of the magnetic field and is commonly expressed as a complex circularly polarized field:

(2) $$B_{1}^{+}\;=\;(B_{x}\;+\;jB_{y})/2$$

This field is critical for spin excitation and efficient signal generation during MRI scans [4,7]. The development of RF coils for ultra-high-field MRI presents significant challenges, particularly due to the shorter electromagnetic wavelength at higher frequencies, which leads to less uniform RF transmission fields (|B_1_|-field) compared to those at lower field strengths. Loop coils remain a state-of-the-art solution because of their simplicity and relatively efficient field performance. However, their ability to produce uniform magnetic fields diminishes as frequency increases [8,9,10].

To address this limitation, transmission line-based structures such as microstrip coils have been proposed [11,12]. Microstrip coils can provide improved field homogeneity at high frequencies. Their dimensions are frequency-dependent, and for 500 MHz (corresponding to 11.74 T), the physical length of the microstrip becomes practical for implementation.

One of the key safety concerns in MRI is the absorption of RF energy by the patient’s tissues, quantified as the specific absorption rate (SAR), measured in watts per kilogram (W/kg). For head imaging, international safety guidelines limit the SAR to a maximum of 3.2 W/kg when using volume transmission coils [13]. At 500 MHz, conventional eight-channel loop coil arrays used for head imaging can exceed this SAR limit—particularly when the input power is scaled to match the |B_1_|-field strength achieved at lower frequencies [14]. This highlights the need for novel coil designs that can achieve uniform field distribution while maintaining the SAR within safe limits.

While microstrip- and loop-based coils have been studied individually at various field strengths, little work has been performed to adapt microstrip structures into elliptical geometries optimized for ultra-high-field systems operating at 500 MHz. Moreover, existing studies typically focus on whole-brain coverage or conventional array geometries, often without addressing SAR limitations specific to ultra-high-field imaging. This work aims to fill that gap by presenting a geometrically compact, lobe-targeted design that not only enhances |B_1_|-field uniformity but also reduces the localized SAR, offering a viable path forward for safe and effective imaging at 11.74 T.

In this work, we present the design of a modified microstrip coil with an elliptical geometry, aimed at generating a more uniform magnetic |B_1_|-field for targeted brain lobe imaging at 11.74 T. Building on this design, a three-channel elliptical microstrip array was developed to improve field homogeneity across individual brain lobes. The decision to focus on individual lobes, rather than whole-brain coverage, stems from concerns about SAR limitations at ultra-high fields. By limiting the imaging region, localized RF energy deposition is reduced, helping to ensure compliance with SAR safety thresholds. The electromagnetic performance of the proposed design is evaluated and compared with that of a conventional loop coil. To verify the proposed design based on simulations, the microstrip array was also fabricated, and bench-top measurements were conducted to confirm its resonance at the target frequency and assess its coupling performance.

## 2. Materials and Methods

The resonance frequency of a straight microstrip depends on the length and width of the conductor, the dielectric constant of the material, and the dielectric height [15]. One issue with straight microstrips is localized propagation of the magnetic field, which fails to cover large areas, which requires the combination of additional microstrips. Previous research has demonstrated the potential to expand microstrip magnetic field coverage by redesigning it into a rectangular loop for 7 T MRI applications [12]. We propose a modification to this concept to suit 11.74 T MRI systems, along with a geometry for the interleaving of three elements to minimize decoupling between elements.

**Figure 1:**
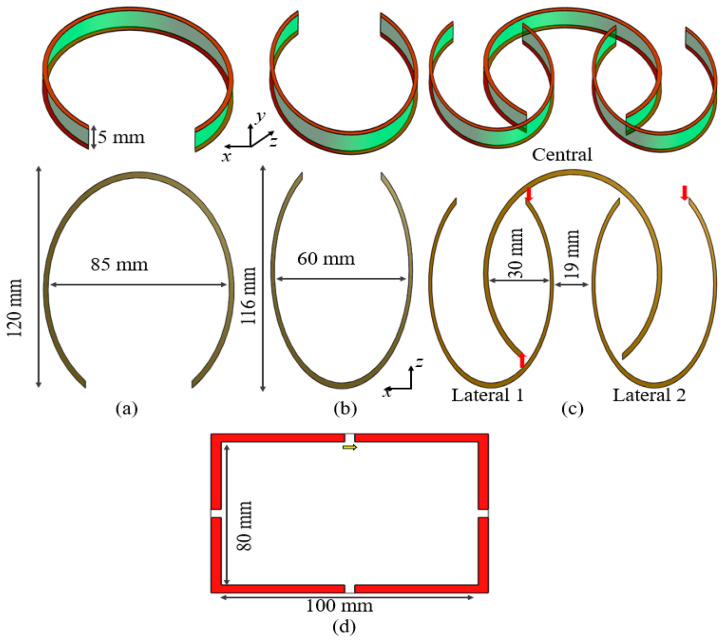
Geometry of the elliptical microstrips: (**a**) center, (**b**) lateral, and (**c**) the combination of three elliptical microstrips. The 3D design is shown in the top row, and the 2D geometry in the bottom row. (**d**) Geometry of the reference coil.

The design of a single-element elliptical microstrip in [Figure 1] consists of two conductor layers: one for current conduction and the other as a ground plane. These conductor layers are separated by a 5 mm height dielectric material selected to be FR4. The proposed three-element array consists of two types of elliptical microstrips. The central microstrip in [Figure 1a] is larger than the two lateral microstrips in [Figure 1b]. The central elliptical microstrip has a large diameter of 120 mm and a small diameter of 85 mm, while the lateral elliptical microstrips have a large diameter of 116 mm and a small diameter of 60 mm. The microstrips have a gap at the end of the strip to avoid forming a complete loop. The placement of this gap allows for interleaving of the smaller elliptical microstrips [Figure 1c]. The central microstrip has a gap of 50 mm, while the lateral microstrips have a gap of 35 mm. The lateral microstrips were placed in the opposite direction from the central microstrip to ensure the fields added up positively. The separation between the lateral elliptical microstrips was 19 mm. The conductor lines for all the microstrips have a thickness of 5 mm. To compare the proposed designs, we also used a conventional loop coil [Figure 1d], with a size of 80 × 100 mm. Figure 1 shows the placement and direction of the voltage’s sources with red arrows.

### 2.1. Simulation Set-Up

**Figure 2:**
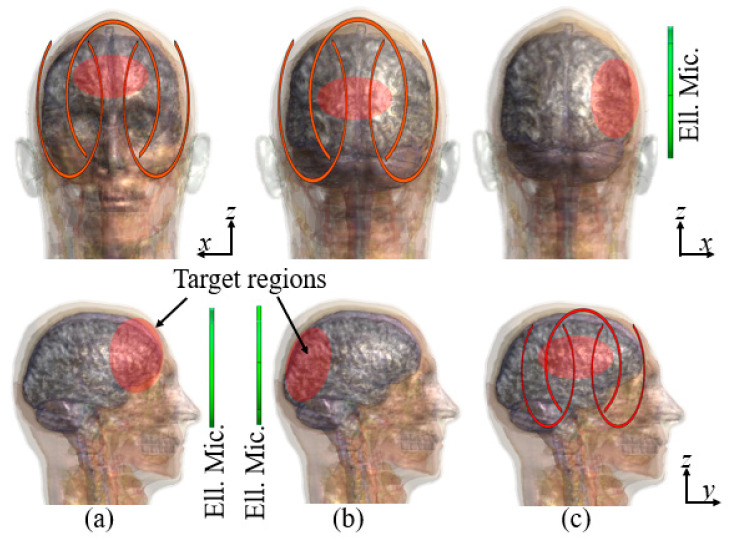
The position of the elliptical microstrips (Ell. Mic.) in (**a**) the frontal lobe, (**b**) occipital lobe, and (**c**) temporal lobe. The top row shows the x–z plane view, and the bottom row shows the y–z plane view. The target region for each elliptical microstrip is indicated with a red area.

Electromagnetic (EM) simulations were performed using commercial FDTD software (Sim4Life by ZMT, www.zmt.swiss (accessed on 25 June 2025), Zurich, Switzerland). The simulations were performed by exciting each of the elliptical microstrips with a gaussian pulse of a central frequency of 500 MHz and a bandwidth of 1 GHz. The voltage source of the central elliptical microstrip was given a 180 degree phase difference in relation to the lateral elliptical microstrips. Simulations were performed by setting all the conductor lines as perfect conductors. To compute the magnetic |B_1_|-field, we used a 3D human model provided by the simulation software DUKE [16]. The model consists of over 100 segmented tissues and organs, each with their respective electrical properties. The three elliptical microstrips were placed around each of the brain lobes—frontal, occipital, and temporal [Figure 2]. The reference loop coil was also placed in the same manner as the elliptical microstrips. The regions of interest within each lobe have been highlighted with red circles in Figure 2 for easier reference.

### 2.2. Analysis Metric

To evaluate the performance of the proposed design, we analysed the uniformity of the |B_1_|-field by computing the coefficient of variation (CV) in the selected region of interest (ROI), indicated by the red circles in Figure 2, as well as globally across the entire selected plane view. The CV is defined by dividing the standard deviation by the mean value. A lower CV value indicates that the field is more uniform. The maximum SAR average of 10 g of tissues was also computed by following the equation:

(3) $${SAR}_{10g}\;=\;\frac{\sigma }{2\rho }E^{2}$$

where *E* is the electric field and *ρ* is the mass density of the tissues (kg/m^3^). Computation of the SAR_10g_ was performed with EM simulation software (Sim4Life Ver. 7.0.1). To compare the performance of each of the elements, the input power was normalized to 1 W.

### 2.3. Bench-Top Development

**Figure 3:**
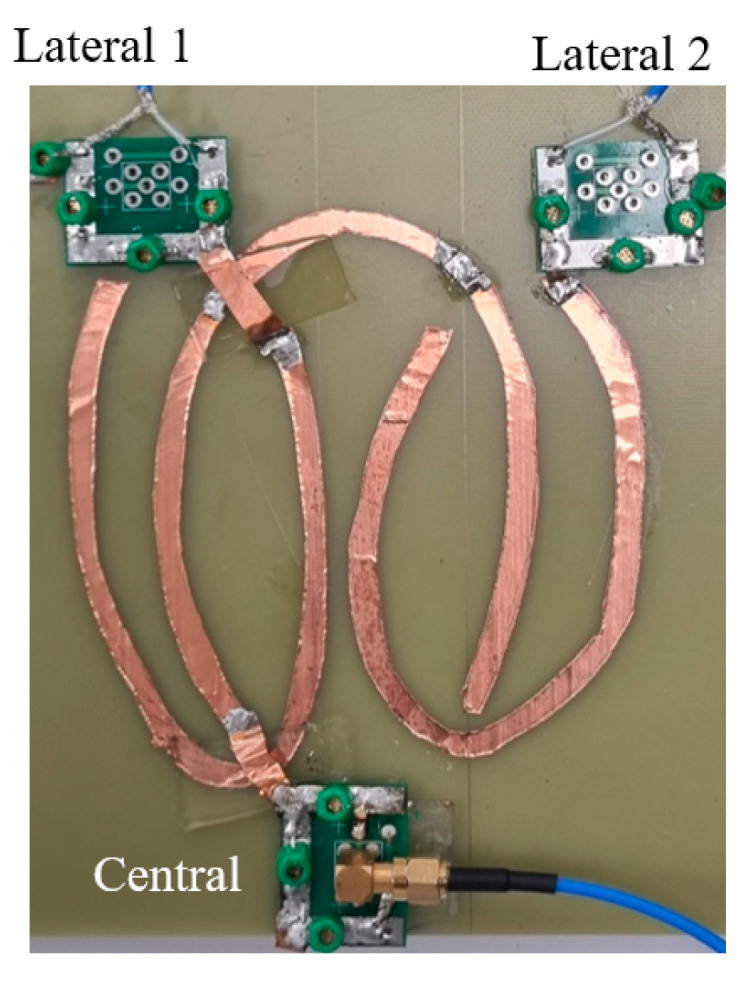
The developed elliptical microstrips with the corresponding matching circuits.

The proposed elliptical microstrips were also fabricated to verify their ability to resonate at 500 MHz. The microstrips were constructed on an FR-4 dielectric substrate using copper tape for the conductor lines, and matching circuits were incorporated to achieve a 50-ohm impedance. In this experiment, only the S-parameters were measured as imaging with the 11.74 T system is still under development. Figure 3 shows a photograph of the fabricated microstrip. The dimensions of the microstrip were 110 mm in length and 70 mm in width, with the copper tape having a width of 5 mm. Unlike the simulations, the FR-4 material was not cut into an elliptical shape, and the ground plane was implemented as a uniform copper sheet.

## 3. Results

**Figure 4:**
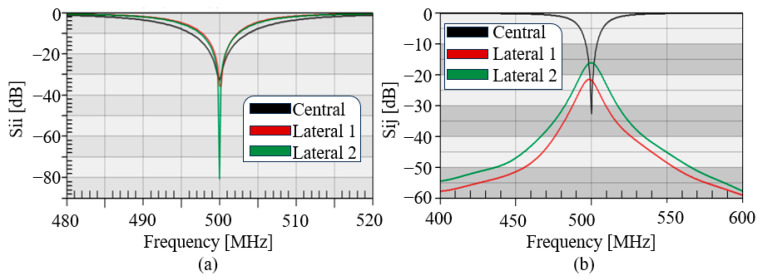
Tuning and matching with (**a**) Sii parameters for the three types of elliptical microstrips. (**b**) Sij parameters for decoupling of the central elliptical microstrip in relation to the lateral elliptical microstrips.

Based on the design geometry of the central and lateral elliptic microstrips, simulations were conducted to determine the frequency response. The scattering Sii parameters for each individual elliptical microstrip are shown, with their tuning frequency at 500 MHz and 50-ohm matching [Figure 4a]. To achieve the target frequency, loading capacitors of 1.2 and 0.5 pF were used for the central and lateral microstrips, respectively. The Sij parameter plots of the central microstrip showed a high decoupling of −18 and −21 dB with respect to the lateral microstrips [Figure 4b].

**Figure 5:**
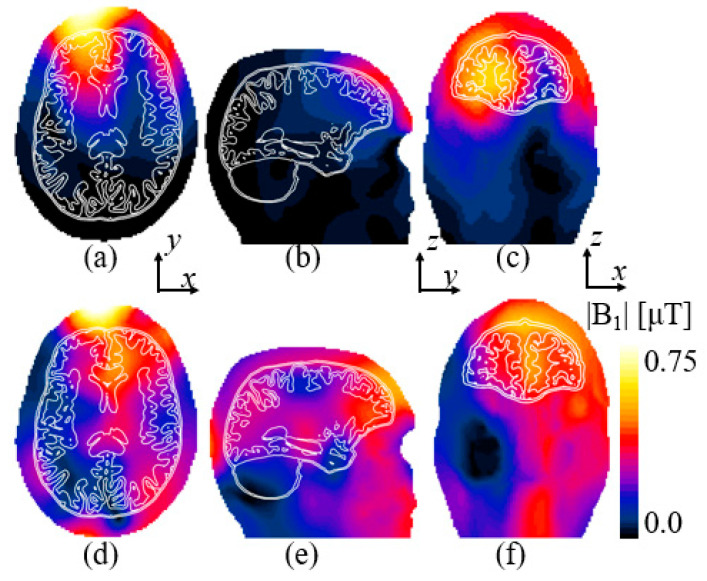
The computed |B_1_|-field in the frontal lobe for the reference coil in (**a**) axial, (**b**) sagittal, and (**c**) coronal views. The |B_1_|-field for the elliptical microstrips in (**d**) axial, (**e**) sagittal, and (**f**) coronal views.

The computed magnetic |B_1_|-field for targeting the frontal lobe based on Figure 2a is displayed in Figure 5. The comparison maps show three types of views, namely axial (x–y axis), sagittal (z–y), and coronal (x–z). The magnetic |B_1_|-field produced by the reference loop coil is displayed in Figure 5a–c, and for the elliptical microstrips, the |B_1_|-field maps are shown in Figure 5d–f. It can be seen that the field map produced by the loop coil has a lower value in the right part of the frontal lobe, whereas the elliptical microstrip shows a more uniform field distribution. The advantage of the proposed method can be seen better in the case of the coronal view, where the area of the frontal lobe has a higher field intensity and uniformity compared to the reference coil. Uniformity of the |B_1_|-field expressed in the CV for each of the views in Figure 5 is summarized in Table 1. Overall, the proposed elliptical microstrip showed an improvement in uniformity of 22.8, 62.2, and 13.88% in the axial, sagittal, and coronal view maps, respectively, with respect to the reference loop coil. The peak SAR_10g_ for the coil and the microstrip was 1.1 and 1.4 W/kg, respectively.

### 3.1. Occipital Lobe

**Figure 6:**
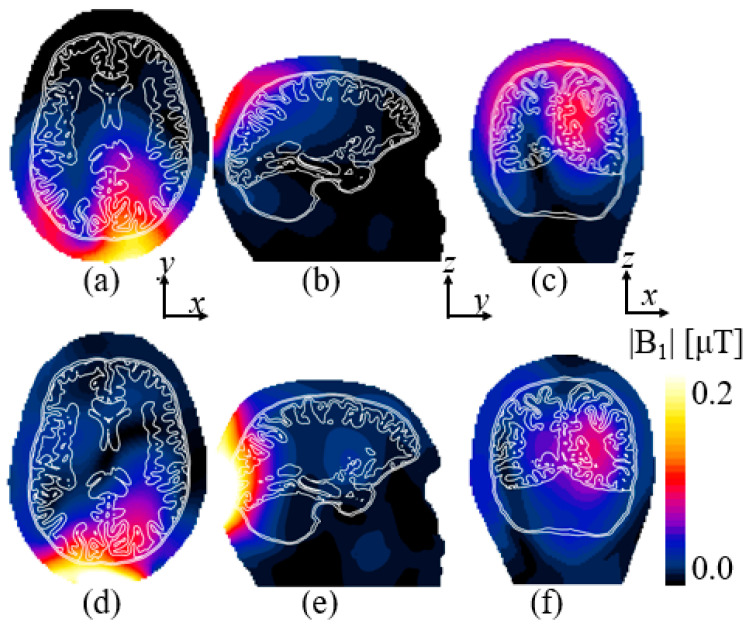
The computed |B_1_|-field in the occipital lobe for the reference coil in (**a**) axial, (**b**) sagittal, and (**c**) coronal views. The |B_1_|-field for the elliptical microstrips in (**d**) axial, (**e**) sagittal, and (**f**) coronal views.

Figure 6 shows the magnetic |B_1_|-field for the occipital lobe computed with the loop and elliptical microstrips. Uniformity of the |B_1_|-field is summarized in Table 1. Improvement in uniformity with respect to the loop coil in the ROI of the occipital lobe was −40, 9.5, and 34%, for the axial, sagittal, and coronal views, respectively. It should be noted that the performance of the elliptical microstrip exhibits a lower performance in the axial view; however, uniformity was improved in the sagittal and coronal views. The coronal view [Figure 6f] shows a more uniform field both in the occipital lobe of the brain and also in the cerebellum. The peak SAR for the coil and elliptical microstrip was 0.8 and 0.9 W/kg, respectively.

### 3.2. Temporal Lobe

**Figure 7:**
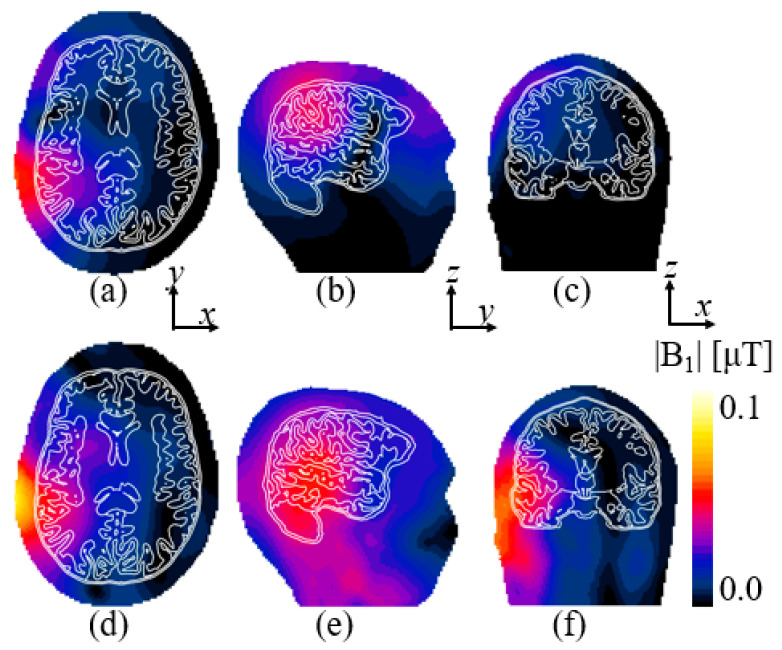
The computed |B_1_|-field in the temporal lobe for the reference coil in (**a**) axial, (**b**) sagittal, and (**c**) coronal views. The |B_1_|-field for the elliptical microstrips in (**d**) axial, (**e**) sagittal, and (**f**) coronal views.

Figure 7 shows the magnetic |B_1_|-field computed with the reference and elliptical microstrips for the right temporal lobe. Uniformity of the |B_1_|-field is summarized in Table 1. Uniformity in the ROI was improved by 32.6, 38.3, and 56% for the axial, sagittal, and coronal views, respectively, with respect to the reference loop coil. The peak SAR_10g_ for the coil and elliptical microstrips was 0.4 and 0.7 W/kg, respectively.

### 3.3. Bench-Top Measurements

**Figure 8:**
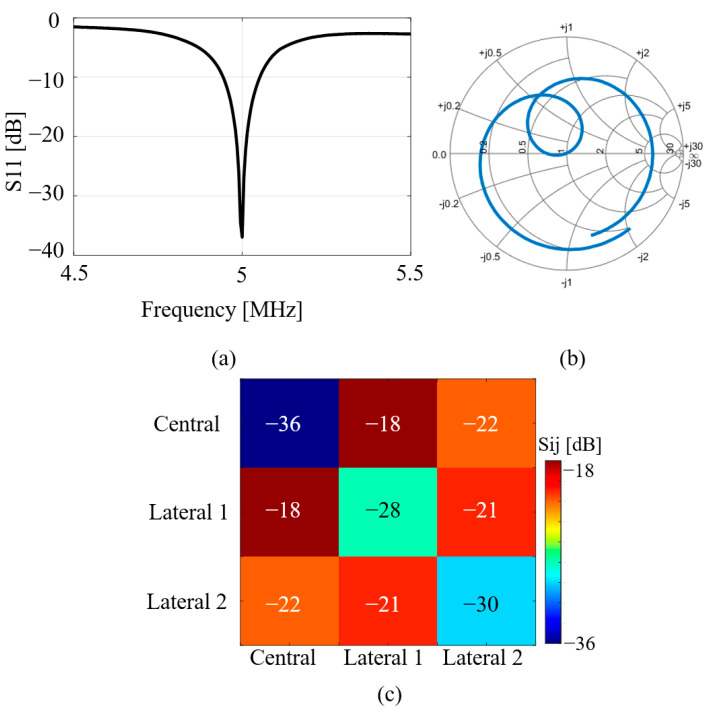
Measurements with the developed elliptical microstrips: (**a**) tuning, (**b**) matching, and (**c**) Sij parameters for decoupling of the central elliptical microstrip in relation to the lateral elliptical microstrips.

Figure 8 presents the results of the S-parameter measurements obtained from the fabricated elliptical microstrips. As shown in Figure 8a, the proposed antenna successfully resonates at the target frequency of 500 MHz, confirming its capability to operate at the intended ultra-high-field strength. Figure 8b illustrates the impedance-matching results, demonstrating that the matching circuits effectively achieved a 50-ohm impedance, which is essential for minimizing signal reflection and maximizing power transfer. In Figure 8c, the measured coupling Sij parameters between the three channels are displayed. The coupling levels observed were consistent with those predicted by the simulations, indicating that the fabricated array maintains the expected electromagnetic behavior in a multi-channel configuration.

## 4. Discussion

We presented the concept of a triple elliptical microstrip array for focused brain lobe imaging using an 11.74 T MRI system [17]. Through electromagnetic simulations, we demonstrated that the modified elliptical microstrip design can produce more uniform magnetic fields compared to conventional loop coils. The proposed design effectively focused the magnetic field into specific brain lobes, resulting in increased field strength in the targeted regions.

Although the peak SAR_10g_ values generated by the elliptical microstrips were slightly higher than those of the reference loop coil, they remained within the safety limits established for head imaging. The triple elliptical microstrip array improved field uniformity in all evaluated cases by 23.76% compared to the loop coil. When considering only the global field distribution, the improvement reached 34.4%, while for localized regions of interest (ROIs), the improvement was approximately 10%.

The simulations were validated through measurements of the fabricated elliptical microstrips, demonstrating that the proposed concept is functional. However, slight deviations in size were observed during fabrication, likely due to the FR-4 dielectric material not being cut into an elliptical shape as in the simulation model. Nevertheless, the resonance frequency and inter-element coupling observed in the measurements closely matched the simulation results. Although the elliptical shape may present some design and fabrication challenges, alternative geometries such as circular or rectangular microstrips could also be considered. The impact of these alternative shapes on performance warrants further investigation.

In this work, we have limited our comparison to a conventional loop coil, although we acknowledge that other advanced coil designs—such as dipole antennas, microstrip arrays, hybrid combinations (e.g., loop-dipole), and RF shimming approaches—are also promising candidates for use in 11.74 T MRI systems [18]. However, since these alternatives are not yet established as standard configurations at this field strength, we chose to benchmark our proposed antenna design against the traditional loop coil for consistency and clarity. Future studies will include comparative analyses with these alternative coil types to further evaluate and optimize their performance at 11.74 T.

We anticipate that this modified microstrip approach can be applied to the future development of 11.74 T MRI scanners for human imaging. This targeted approach facilitates high-resolution imaging of localized brain regions while minimizing the risks associated with elevated SAR levels.

## 5. Conclusions

In this study, we proposed and evaluated a novel triple elliptical microstrip array designed for targeted brain lobe imaging at 11.74 T MRI. Through electromagnetic simulations and preliminary bench-top validation, we demonstrated that the modified elliptical microstrip geometry provides improved magnetic field uniformity compared to traditional loop coils. The array successfully focused the |B_1_|-field into specific brain lobes, achieving stronger field coverage in the regions of interest.

While the SAR_10g_ values produced by the microstrip array were slightly higher than those from loop coils, they remained within international safety guidelines for head imaging. The design yielded a 23.76% improvement in overall field uniformity, with localized improvements of up to 10% and global uniformity gains of 34.4%. These results indicate that elliptical microstrip arrays are a promising alternative for ultra-high-field MRI applications, especially in scenarios where focused imaging and SAR control are critical. Future work will include extended comparisons with other coil designs (e.g., dipoles and straight microstrips), and in-system imaging once the 11.74 T scanner becomes fully operational.

## Figures and Tables

**Table 1:** Uniformity of the |B_1_|-field [CV].

View	Frontal Lobe		Occipital Lobe		Temporal Lobe	
Coil Type	Loop Coil	Elliptical Microstrip	Loop Coil	Elliptical Microstrip	Loop Coil	Elliptical Microstrip
Global Axial	1.10	0.49	1.05	1.17	0.84	0.86
ROI Axial	0.57	0.44	0.35	0.49	0.46	0.31
Global Sagittal	1.3	0.39	1.3	1.6	0.62	0.35
ROI Sagittal	0.45	0.17	0.74	0.67	0.47	0.29
Global Coronal	0.87	0.36	0.80	0.51	1.16	0.89
ROI Coronal	0.16	0.31	0.44	0.29	0.50	0.22
Global Axial	1.10	0.49	1.05	1.17	0.84	0.86

## Data Availability

Upon request to the corresponding author.
